# Is the period of austerity in the UK associated with increased rates of adverse birth outcomes?

**DOI:** 10.1093/eurpub/ckae154

**Published:** 2024-11-01

**Authors:** Rachael Watson, David Walsh, Sonya Scott, Jade Carruthers, Lynda Fenton, Gerry McCartney, Emily Moore

**Affiliations:** Public Health Scotland, Edinburgh, United Kingdom; School of Health & Wellbeing, University of Glasgow, Glasgow G12 8TB, United Kingdom; Public Health Scotland, Edinburgh, United Kingdom; Public Health Scotland, Edinburgh, United Kingdom; Public Health Scotland, Edinburgh, United Kingdom; School of Social and Political Sciences, University of Glasgow, Glasgow, United Kingdom; Public Health Scotland, Edinburgh, United Kingdom

## Abstract

Hugely concerning changes to health outcomes have been observed in the UK since the early 2010s, including reductions in life expectancy and widening of inequalities. These have been attributed to UK Government ‘austerity’ policies which have profoundly affected poorer populations. Studies in mainland Europe have shown associations between austerity and increases in adverse birth outcomes such as low birthweight (LBW). The aim here was to establish whether the period of UK austerity was also associated with higher risks of such outcomes. We analysed all live births in Scotland between 1981 and 2019 (*n* = 2.3 million), examining outcomes of LBW, preterm birth (PB) and small-for-gestational-age (SGA). Descriptive trend analyses, segmented regression (to identify changes in trends) and logistic regression modelling (to compare risk of outcomes between time periods) were undertaken, stratified by infant sex and quintiles of socioeconomic deprivation. There were marked increases in LBW and PB rates in the austerity period, particularly in the most deprived areas. However, rates of SGA decreased, suggesting prematurity as the main driver of LBW rather than intrauterine growth restriction. The regression analyses confirmed these results: trends in LBW and PB changed within 1–3 years of the period in which austerity was first implemented, and that period was associated with higher risk of such outcomes in adjusted models. The results add to the European evidence base of worsening birth outcomes associated with austerity-related economic adversity. The newly elected UK government needs to understand the causes of these changes, and the future implications for child and adult health.

## Introduction

There have been marked, adverse, changes in different population health outcomes since the early 2010s across all parts of the UK: increasing death rates among socioeconomically deprived groups (and stalled improvements in mortality rates across the whole population), declining healthy life expectancy, and poorer mental health [1]. These changes, which predate the COVID-19 pandemic but have been exacerbated by it, have been largely attributed to UK Government ‘austerity’ measures introduced in 2010 that have particularly impacted on the poorest and most vulnerable populations through loss of income (social security) and important services [1–3]. The causal pathways involved, including the inability to obtain important material goods and services, the impact of increased level of stress on poor mental health and risk of chronic disease, and changes in health-damaging ‘coping mechanisms’ (behavioural responses—use of tobacco, alcohol, drugs etc), are well understood [4–6]. Further details of the austerity policies and the evidence of their impact are included in Box A1 in the Supplementary appendix.

Adverse birth outcomes such as low birth weight (LBW) and preterm birth (PB) have also been shown to be associated with poverty and other ‘economic stressors’ [7–9], with the effects best explained by a similar combination of biological (stress-related pathways impacting on placental function and foetal development) and behavioural factors [8, 10]. Consequently, socioeconomic inequalities in birth outcomes is clear and well documented [9, 11]. For example, a systematic review and meta analyses of UK and Irish studies showed that mothers of lower socioeconomic position (SEP) were ∼40% more likely to have both a preterm and low birthweight baby than those of higher SEP [11].

As a composite measure of both birthweight and gestational age, ‘small for gestational age’ (SGA) is another birth outcome of potential interest. Trends and socioeconomic patterns in all three outcomes have been shown previously to be similar in Scotland (e.g. with a similar association with SEP) [12], although trends in particular outcomes can vary in time and place. All three outcomes share a number of important risk factors including a wide range of socioeconomic characteristics (e.g. SEP, poverty, deprivation) as well as ethnicity, maternal smoking, a history of previous adverse birth events (LBW, PB, SGA), maternal and paternal characteristics, and more [13–15].

Given the importance of socioeconomic exposures, a number of European studies have also highlighted associations between periods of austerity and/or economic crises and greater risks of such adverse birth outcomes [16–23]. However, to our knowledge, this has not been examined in a UK context. Given that all three outcomes are in turn associated with poorer outcomes later in life [7, 9, 24], and that austerity policies are still debated at UK government level [25, 26], this is an important issue to understand. The aim here, therefore, was to examine the association between the time period in which austerity was first implemented in the UK and risk of these three birth outcomes since then. We focus on Scotland, and compare risks across levels of socioeconomic deprivation. Specifically, we sought to:

1. identify any break points (changes) in trends in the three birth outcomes;
2. assess whether the period of austerity is independently associated with a greater risk of the three outcomes in the Scottish population while controlling for any relevant confounders.

## Methods

### Data sources

We used Public Health Scotland’s Maternity Inpatient and Day Case Scottish Morbidity Record dataset (SMR02) which records all hospital-based obstetric events in Scotland. We extracted all live births in the period 1981–2019 (*n* = 2 273 548).

To provide relevant context to the trends, we additionally examined child poverty rates (defined as the percentage of children living in relative poverty after housing costs): These data were supplied by the Scottish Parliament Information Centre (SPICe) [27]. Child poverty is a more relevant measure for the population being studied than poverty at all ages, and it has been shown to be important in understanding the effects of austerity policies on child health outcomes [17]. However, as data for Scotland were only available from 1994 onwards, we used data for the whole of the UK for the years 1981–93.

### Outcome variables

We examined three birth outcomes: low birthweight babies; premature births; and SGA babies. Full definitions of all three are included in Box A2 in the Supplementary appendix.

### Statistical analyses

#### Descriptive analyses

We examined trends in the three outcomes over the time period, stratified by sex of baby, and socioeconomic deprivation quintile. Following guidance, the latter were based on different versions of the Scottish Index of Multiple Deprivation (SIMD) for the period 1996–2019, and the Carstairs-Morris index for the years 1981–95 [28]. Both are area-based measures of relative deprivation. The SIMD is a much more comprehensive measure, derived at a much smaller spatial scale; however, historical data are limited. Carstairs-Morris (based on a much more limited set of variables and calculated for larger neighbourhoods) is available back to the early 1980s and is, therefore, used for analyses of longer term trends.

For clearer interpretation of trends, lines were smoothed using three-year rolling averages (in the descriptive analyses only). Trends by deprivation quintile were compared with trends in relative child poverty.

#### Segmented regression

To identify break points in the birth outcomes trends, we fitted segmented logistic regression models to the data. Models were fitted using the ‘ljr’ package of R [29] which estimated the location of any breakpoint (details of the precise methods used can be found elsewhere [30]). As logistic regression estimates the log-odds that an outcome will happen, the percentage of live births was, therefore, log-transformed. Further details including the modelling equation used are included within the supplementary material (Supplementary Box A1).

#### Logistic regression

A directed acyclic graph (DAG) was first developed as the basis for the selection of variables for the logistic regression modelling. This was based on searches of the literature, identification of relevant risk factors, and an understanding of the causal pathways linking the exposure (austerity) and outcomes (LBW, PB, and SGA). The DAG is shown in Fig. A1 in the Supplementary Material. A number of risk factors for adverse birth outcomes were theorized to lie on the causal pathway between exposure and outcome, and thus were not included in the final models. These included maternal age (as women may delay pregnancy for economic reasons [31]), unhealthy commodities such as smoking (as these are known to be responses to economic adversity [10]), and maternal morbidity (also linked to poverty). The remaining variables considered for inclusion were: Previous LBW/PB/SGA pregnancy; ethnicity (reflecting the impact of racism and discrimination [32]); and parental (here maternal) height. In the final models, we included maternal height only. This was because of substantial missing ethnicity data, and because the inclusion of previous LBW/PB/SGA births could potentially introduce a bias: the austerity period is from 2010 onwards and given the average (short) gap between births, women would be more likely to have a previous LBW/PB/SGA birth recorded early on in the austerity period (e.g. in 2011 or 2012) than they would later (e.g. in 2018 and 2019) (as the ‘look-back’ period becomes longer the further away from 2010 we get, and thus effects are more likely to be ‘adjusted away’ immediately after 2010 than later on).

**Figure 1.**
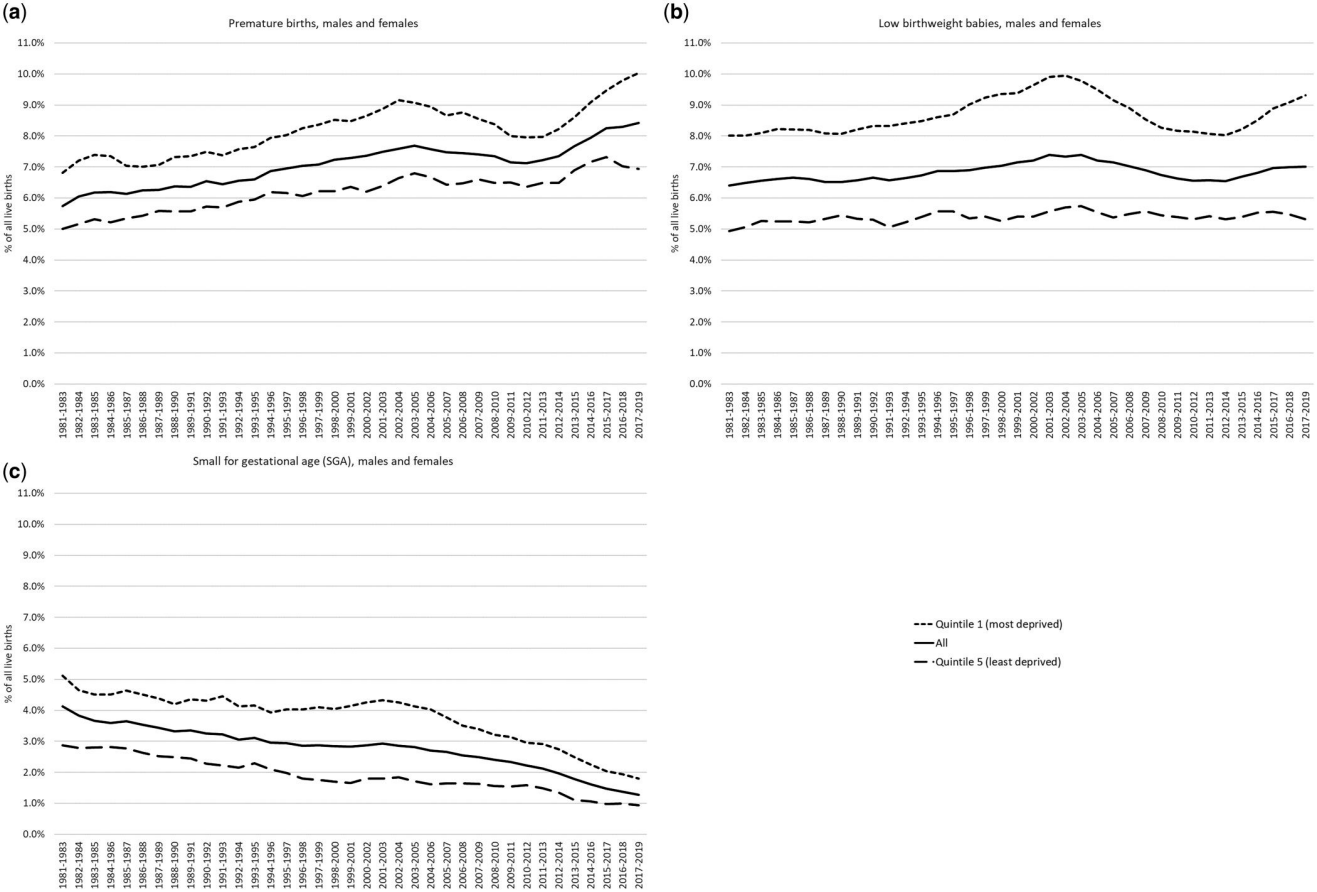
Trends in (a) premature births, (b) low birthweight babies, and (c) small for gestational age babies, Scotland and its most and least deprived quintiles, 1981/83–2017/19.

In the models, we compared the probability of a baby being born LBW/PB/SGA (in separate models, not combined) in the austerity period compared with earlier periods. To ensure a best fit of the data in the models, the austerity period and the definition and number of ‘earlier periods’ were based on the results of segmented regression analyses and the identification of break points in the trends (for the whole population). As described in more detail in the results section below, those post-austerity-implementation breakpoints were 2010 for SGA, 2011 for PB and 2012 for LBW. For LBW and PB, three time periods were used; for SGA only two. The parameters in the models were: pre-austerity slope(s); change in slope after the austerity breakpoint; change in slope after the earlier (pre-2010) breakpoint (LBW and PB only); and maternal height. The full equation for the modelling is shown in Box A2 in the Supplementary Material.

#### Sensitivity analyses

Analyses were restricted to live *singleton* births (rather than just all live births), and to live singleton births excluding planned Caesarean sections. Additional analyses of SGA (e.g. examining live births and still births) were also undertaken. In all cases, results were very similar (see Results section).

## Results

Figure 1 shows trends in the three birth outcomes, comparing the whole population with those living in the 20% least and most socioeconomically deprived neighbourhoods of Scotland. Fig. A2 in the Supplementary Material shows trends for all five quintiles. Increases in rates of LBW and PB from the early 2010s can be seen for all births, but particularly so among those in the most deprived quintile. However, this is not the case for SGA births, rates of which declined through most of the period. Trends were broadly similar for males and females (Figs A3 and A4 in the Supplementary Material).

**Figure 2.**
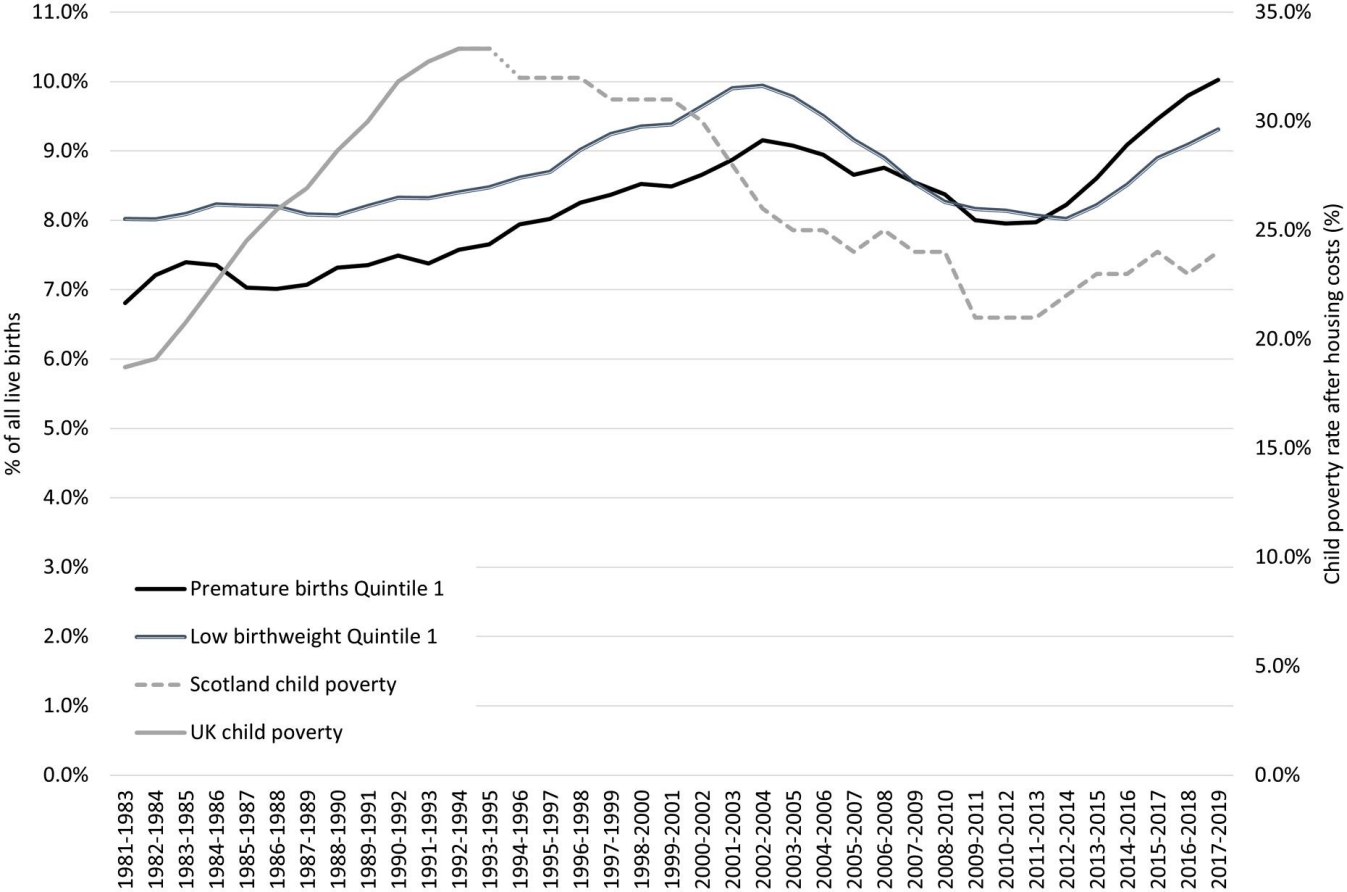
Premature and low birthweight births (most deprived quintile) and child poverty, Scotland 1981–2019.

Figure 1 also highlights notable increases in rates in the most deprived quintile around the mid-1990s (with Supplementary Fig. A2 emphasizing the clear distinction between this quintile and the other four). This is particularly evident for LBW, but is also seen for the other two outcomes, including SGA.

Figure 2 shows that the increase in LBW and PB in the most deprived quintile from the early 2010s corresponds with an increase in levels of child poverty in that period in Scotland. Previous increases in rates of LBW/PB from the mid-1980s to the early 2000s also overlapped with a period of increasing child poverty (albeit interpretation is made difficult by the unavailability of Scottish poverty data for some of that period). To a lesser extent this is also true of rates of SGA births in the most deprived quintile in the mid-to-late 1990s.

Child poverty rates in Scotland have broadly followed the same trajectory as rates for the whole of the UK; however, rates have been lower in Scotland since the early-to-mid 2000s (Fig. A5 in the Supplementary Material).

Sensitivity analyses showed that the increases in rates of PB and LBW (particularly in the most deprived quintile) were also observed when the analyses were restricted to live singleton births only, and to live singleton births excluding planned Caesarean sections (Figs A6 and A7 in the Supplementary Material).

Table 1 lists the estimated breakpoints from the segmented regression analyses. Plots are included within the online appendix (Supplementary Figs A8–A11). Confirming the impression of the descriptive analyses shown in Fig. 1, breakpoints for (worsening) changes in trends in the period 2010 onwards were identified for LBW and PB [including in both cases for male and female births in the most deprived 20% of areas (with those of males preceding those of females)], but not for SGA. These breakpoints in the relevant period were all around 2011–13 (i.e. within 3 years of the introduction of austerity policies).

**Table 1.** Estimated breakpoints from segmented regression analyses for three birth outcomes, stratified by sex and deprivation quintile (most and least deprived)^a^

Birth outcome	Stratification by deprivation and sex	Estimated breakpoints
Premature birth		1983.0 ↑ 2004.6 ↓ **2011.1 ↑**
	Quintile 1 (most deprived), male	2001.0 ↑ 2002.1 ↓ **2011.0 ↑**
	Quintile 5 (least deprived), male	1994.0 ↓
	Quintile 1 (most deprived), female	2004.0 ↓ **2012.8 ↑**
	Quintile 5 (least deprived), female	2016.1 ↓
Low birthweight		1993.0 ↑ 2003.0 ↓ **2012.0 ↑**
	Quintile 1 (most deprived), male	1993.0 ↑ 2002.8 ↓ **2010.0 ↑**
	Quintile 5 (least deprived), male	2004.0 ↓
	Quintile 1 (most deprived), female	2003.6 ↓ **2012.9 ↑**
	Quintile 5 (least deprived), female	*No breakpoint identified*
Small for gestational age		1983.7 ↑ 2009.7 ↓
	Quintile 1 (most deprived), male	1983.6 ↑ 1999.9 ↑ 2001.1 ↓
	Quintile 5 (least deprived), male	2011.8 ↓
	Quintile 1 (most deprived), female	1982.4 ↓ 2005.4 ↓
	Quintile 5 (least deprived), female	*No breakpoint identified*

a Bold font indicates breakpoints which mark an increase in rate in the period from 2010 onwards; up arrows (↑) next to breakpoints indicate a less favourable change in slope (i.e. increased rates or reduced improvement) in subsequent period; down arrows (↓) indicate more favourable change in slope.

It is also notable in Table 1 that there are more breakpoints in trends in the most deprived quintile compared to the least deprived quintile.

Table 2 presents the results of the final logistic modelling analyses. Odds ratios comparing risks of the three outcomes in the period after 2010 (defined by the breakpoints identified in the segmented regression) compared to earlier period(s) are shown for all three outcomes separately, stratified by sex and socioeconomic deprivation (again, most and least deprived quintiles). These results confirm those of the segmented regression, showing notably increased risks of LBW and PB, but not SGA, in the austerity period. For example, for PB there is an odds ratio of 1.07 for male infants born to mothers living in the most deprived quintile after 2011 compared to and odds ratio of 0.96 between 2005 and 2011. In other words, the odds of such an outcome increases by 7% each year post 2012, having decreased by 4% each year in the previous 6 years. Similarly, for PB and LBW, the coefficients for the initial (pre-changes) periods are consistently greater than 1, reflecting the steady increases in rates of both outcomes prior to the later changes (i.e. the decreases from the early-to-mid 2000s, and increases post 2010).

**Table 2.** Results of logistic regression modelling with outcomes of (a) LBW, (b) PB, and (c) SGA babies

			Adjusted		Unadjusted	
Outcome	Population	Sex/parameter	OR	(95% CI)	OR	(95% CI)
**(a) Low birthweight (2 breakpoints)**	**All**	**Both sexes**				
		*β*_1_ (initial slope 1981–2002)	1.012	(1.011–1.013)	1.007	(1.007–1.008)
		*δ*_1_ (change in slope after 2003)	0.984	(0.980–0.987)	0.980	(0.977–0.982)
		*δ*_2_ (change in slope after 2012)	1.025	(1.018–1.031)	1.027	(1.021–1.033)
		*β*_2_ (height)	0.956	(0.955–0.957)		
		**Male**				
		*β*_1_ (initial slope 1981–2002)	1.012	(1.011–1.014)	1.008	(1.007–1.009)
		*δ*_1_ (change in slope after 2003)	0.981	(0.977–0.986)	0.977	(0.973–0.981)
		*δ*_2_ (change in slope after 2012)	1.026	(1.017–1.036)	1.027	(1.018–1.036)
		*β*_2_ (height)	0.956	(0.955–0.957)		
		**Female**				
		*β*_1_ (initial slope 1981–2002)	1.012	(1.010–1.013)	1.007	(1.005–1.008)
		*δ*_1_ (change in slope after 2003)	0.986	(0.981–0.990)	0.982	(0.978–0.986)
		*δ*_2_ (change in slope after 2012)	1.024	(1.015–1.033)	1.026	(1.018–1.035)
		*β*_2_ (height)	0.956	(0.955–0.957)		
	**Quintile 1 (most deprived)**	**Both sexes**				
		*β*_1_ (initial slope 1981–2002)	1.016	(1.014–1.018)	1.009	(1.007–1.011)
		*δ*_1_ (change in slope after 2003)	0.970	(0.964–0.976)	0.974	(0.968–0.980)
		*δ*_2_ (change in slope after 2012)	1.047	(1.035–1.059)	1.047	(1.035–1.059)
		*β*_2_ (height)	0.960	(0.958–0.961)		
		**Male**				
		*β*_1_ (initial slope 1981–2002)	1.015	(1.013–1.018)	1.008	(1.005–1.010)
		*δ*_1_ (change in slope after 2003)	0.972	(0.963–0.980)	0.976	(0.967–0.984)
		*δ*_2_ (change in slope after 2012)	1.043	(1.026–1.061)	1.044	(1.026–1.062)
		*β*_2_ (height)	0.960	(0.957–0.962)		
		**Female**				
		*β*_1_ (initial slope 1981–2002)	1.017	(1.014–1.020)	1.010	(1.007–1.012)
		*δ*_1_ (change in slope after 2003)	0.969	(0.961–0.977)	0.973	(0.965–0.981)
		*δ*_2_ (change in slope after 2012)	1.050	(1.033–1.067)	1.050	(1.033–1.067)
		*β*_2_ (height)	0.960	(0.958–0.962)		
	**Quintile 5 (least deprived)**	**Both sexes**				
		*β*_1_ (initial slope 1981–2002)	1.008	(1.006–1.011)	1.003	(1.001–1.006)
		*δ*_1_ (change in slope after 2003)	0.995	(0.986–1.003)	0.996	(0.987–1.005)
		*δ*_2_ (change in slope after 2012)	1.007	(0.989–1.025)	1.006	(0.988–1.024)
		*β*_2_ (height)	0.960	(0.958–0.962)		
		**Male**				
		*β*_1_ (initial slope 1981–2002)	1.009	(1.005–1.013)	1.004	(1.000–1.007)
		*δ*_1_ (change in slope after 2003)	0.989	(0.976–1.002)	0.991	(0.978–1.004)
		*δ*_2_ (change in slope after 2012)	1.016	(0.990–1.043)	1.015	(0.989–1.041)
		*β*_2_ (height)	0.959	(0.956–0.963)		
		**Female**				
		*β*_1_ (initial slope 1981–2002)	1.008	(1.004–1.012)	1.003	(0.999–1.007)
		*δ*_1_ (change in slope after 2003)	0.999	(0.987–1.012)	1.001	(0.989–1.013)
		*δ*_2_ (change in slope after 2012)	0.999	(0.975–1.023)	0.998	(0.974–1.022)
		*β*_2_ (height)	0.961	(0.958–0.964)		
**(b) Premature birth (2 breakpoints)**	**All**	**Both sexes**				
		*β*_1_ (initial slope 1981–2004)	1.015	(1.014–1.016)	1.013	(1.012–1.014)
		*δ*_1_ (change in slope after 2005)	0.976	(0.972–0.980)	0.971	(0.967–0.975)
		*δ*_2_ (change in slope after 2011)	1.043	(1.036–1.050)	1.046	(1.039–1.053)
		*β*_2_ (height)	0.980	(0.979–0.981)		
		**Male**				
		*β*_1_ (initial slope 1981–2004)	1.015	(1.014–1.017)	1.013	(1.012–1.015)
		*δ*_1_ (change in slope after 2005)	0.971	(0.965–0.977)	0.967	(0.961–0.972)
		*δ*_2_ (change in slope after 2011)	1.049	(1.039–1.059)	1.051	(1.042–1.060)
		*β*_2_ (height)	0.979	(0.978–0.980)		
		**Female**				
		*β*_1_ (initial slope 1981–2004)	1.015	(1.014–1.016)	1.013	(1.012–1.014)
		*δ*_1_ (change in slope after 2005)	0.982	(0.975–0.988)	0.976	(0.971–0.982)
		*δ*_2_ (change in slope after 2011)	1.036	(1.025–1.046)	1.039	(1.030–1.049)
		*β*_2_ (height)	0.980	(0.979–0.982)		
	**Quintile 1 (most deprived)**	**Both sexes**				
		*β*_1_ (initial slope 1981–2004)	1.016	(1.014–1.017)	1.014	(1.012–1.016)
		*δ*_1_ (change in slope after 2005)	0.968	(0.959–0.976)	0.960	(0.953–0.967)
		*δ*_2_ (change in slope after 2011)	1.061	(1.047–1.075)	1.069	(1.057–1.082)
		*β*_2_ (height)	0.983	(0.981–0.985)		
		**Male**				
		*β*_1_ (initial slope 1981–2004)	1.016	(1.014–1.019)	1.015	(1.012–1.017)
		*δ*_1_ (change in slope after 2005)	0.963	(0.952–0.975)	0.957	(0.947–0.967)
		*δ*_2_ (change in slope after 2011)	1.065	(1.046–1.085)	1.072	(1.055–1.090)
		*β*_2_ (height)	0.982	(0.980–0.985)		
		**Female**				
		*β*_1_ (initial slope 1981–2004)	1.015	(1.012–1.017)	1.013	(1.011–1.015)
		*δ*_1_ (change in slope after 2005)	0.973	(0.961–0.985)	0.964	(0.953–0.974)
		*δ*_2_ (change in slope after 2011)	1.055	(1.035–1.076)	1.065	(1.047–1.084)
		*β*_2_ (height)	0.984	(0.981–0.986)		
	**Quintile 5 (least deprived)**	**Both sexes**				
		*β*_1_ (initial slope 1981–2004)	1.015	(1.013–1.018)	1.012	(1.010–1.015)
		*δ*_1_ (change in slope after 2005)	0.980	(0.969–0.991)	0.981	(0.971–0.991)
		*δ*_2_ (change in slope after 2011)	1.026	(1.007–1.045)	1.021	(1.004–1.039)
		*β*_2_ (height)	0.981	(0.979–0.984)		
		**Male**				
		*β*_1_ (initial slope 1981–2004)	1.014	(1.010–1.017)	1.011	(1.008–1.014)
		*δ*_1_ (change in slope after 2005)	0.977	(0.961–0.992)	0.978	(0.964–0.992)
		*δ*_2_ (change in slope after 2011)	1.035	(1.009–1.061)	1.031	(1.007–1.055)
		*β*_2_ (height)	0.979	(0.976–0.982)		
		**Female**				
			1.017	(1.013–1.021)	1.015	(1.011–1.018)
		*δ*_1_ (change in slope after 2005)	0.984	(0.967–1.001)	0.985	(0.970–1.000)
		*δ*_2_ (change in slope after 2011)	1.016	(0.989–1.043)	1.011	(0.987–1.036)
		*β*_2_ (height)	0.984	(0.981–0.987)		
**(c) Small for gestational age (1 breakpoint)**	**All**	**Both sexes**				
		*β*_1_ (initial slope 1981–2009)	0.990	(0.989–0.991)	0.982	(0.981–0.983)
		*δ* (change in slope after 2010)	0.941	(0.935–0.947)	0.941	(0.935–0.946)
		*β*_2_ (height)	0.936	(0.934–0.937)		
		**Male**				
		*β*_1_ (initial slope 1981–2009)	0.990	(0.989–0.992)	0.983	(0.982–0.984)
		*δ* (change in slope after 2010)	0.944	(0.936–0.952)	0.942	(0.934–0.949)
		*β*_2_ (height)	0.935	(0.933–0.937)		
		**Female**				
		*β*_1_ (initial slope 1981–2009)	0.989	(0.988–0.990)	0.982	(0.980–0.983)
		*δ* (change in slope after 2010)	0.938	(0.931–0.946)	0.940	(0.933–0.947)
		*β*_2_ (height)	0.936	(0.934–0.938)		
	**Quintile 1 (most deprived)**	**Both sexes**				
		*β*_1_ (initial slope 1981–2009)	0.996	(0.994–0.998)	0.987	(0.986–0.989)
		*δ* (change in slope after 2010)	0.927	(0.917–0.936)	0.929	(0.920–0.938)
		*β*_2_ (height)	0.942	(0.939–0.944)		
		**Male**				
		*β*_1_ (initial slope 1981–2009)	0.995	(0.993–0.998)	0.987	(0.985–0.990)
		*δ* (change in slope after 2010)	0.931	(0.917–0.946)	0.929	(0.916–0.942)
		*β*_2_ (height)	0.941	(0.937–0.944)		
		**Female**				
		*β*_1_ (initial slope 1981–2009)	0.997	(0.994–0.999)	0.987	(0.985–0.989)
		*δ* (change in slope after 2010)	0.922	(0.909–0.936)	0.929	(0.916–0.942)
		*β*_2_ (height)	0.943	(0.939–0.946)		
	**Quintile 5 (least deprived)**	**Both sexes**				
		*β*_1_ (initial slope 1981–2009)	0.981	(0.979–0.984)	0.975	(0.973–0.978)
		*δ* (change in slope after 2010)	0.973	(0.956–0.990)	0.969	(0.953–0.985)
		*β*_2_ (height)	0.935	(0.931–0.939)		
		**Male**				
		*β*_1_ (initial slope 1981–2009)	0.984	(0.979–0.988)	0.978	(0.974–0.982)
		*δ* (change in slope after 2010)	0.959	(0.934–0.984)	0.954	(0.931–0.977)
		*β*_2_ (height)	0.938	(0.932–0.943)		
		**Female**				
		*β*_1_ (initial slope 1981–2009)	0.979	(0.975–0.984)	0.973	(0.969–0.976)
		*δ* (change in slope after 2010)	0.985	(0.962–1.009)	0.983	(0.961–1.006)
		*β*_2_ (height)	0.932	(0.927–0.937)		

Odds ratios tend to be slightly higher for male infants. The inclusion of maternal height in the adjusted models attenuates the risk very slightly in most models.

## Discussion

### Overall findings and implications

The introduction of UK Government austerity policies from 2010 onwards and increased child poverty coincided with increases in rates of preterm and low birthweight births, particularly among those living in the most socioeconomically deprived neighbourhoods of Scotland. This is confirmed by modelling analysis showing a greater risk of such outcomes compared with earlier periods. However, this was not the case for rates of SGA births, the trends for which were quite different.

As we discuss further below, this adds to an increasingly large evidence base of the adverse impact of economic conditions, and related policy choices, on birth outcome trends in Europe. As LBW and PB are well-known risk factors for other childhood and adulthood negative health outcomes [7, 9], the effects of these adverse trends are likely to be observed for some time. When viewed alongside the extensive evidence of the negative impact of austerity policies (particularly in the UK) on a wide range of social and health outcomes including food bank reliance, homelessness, poor mental health and mortality increases [1], the policy implications in terms of the urgent need to avoid future austerity are obvious.

The different trends in SGA compared with LBW and PB—decreasing rather than increasing in the period of interest—suggest that the increase in LBW has been driven by the increase in PB (i.e. babies having less time to grow before being born), given that there has not been a corresponding increase in babies being born lighter than we would have expected given their gestational age. In addition, the long-term downward trend in SGA babies has been matched by an upward trend in large for gestational age babies in Scotland, suggesting a rightward shift in the population distribution of birthweight [33]. This is also linked to increases in maternal body mass index (BMI) over time [33], and high maternal BMI is associated with a lower risk of a SGA birth [34]. This may be masking any impact of austerity on relative foetal growth (as current growth norms and cut-offs are based on a 1990s reference population, and a shift since then in the overall distribution of birthweight to heavier babies could mask an increasing proportion of babies relatively small by more recent norms), and may partially explain why an increase in rates in SGA births occurred in more deprived areas at a time of high relative poverty in the mid-1990s, but not in the period since 2010.

Given this complexity, an important implication for clinicians, epidemiologists and policymakers alike is the need to avoid examining trends in SGA in isolation from the other important, related, birth outcomes.

### Strengths and weaknesses

A number of limitations of the analyses are acknowledged. In particular, the logistic regression models were limited in the number of covariates that could be included. In some cases, this was entirely justified as a number of known risk factors for adverse birth outcomes including age of mother, maternal smoking, and maternal health conditions lie on the causal pathway between the exposure and outcomes, and their inclusion would thus have represented overadjustment. However, ethnicity—an important risk factor reflecting the impact of discrimination and structural racism [32]—could not be included because of historical recording deficiencies in the data set. In addition, we did not include previous LBW/PB/SGA because of the time-dependent bias this could have introduced (as discussed in the Methods section), and for reasons of data availability, we had to combine different measures of area deprivation over the time period. Similarly, child poverty data for Scotland were not available pre-1994; however, as Supplementary Fig. A5 shows, although rates have been consistently lower in Scotland since the early-to-mid 2000s, the direction of trends in both Scotland and the UK has been broadly similar, and thus it is unlikely that this will have affected the results in any meaningful way.

Key strengths of the work include: the use of a comprehensive data set covering the entire resident population of Scotland over nearly four decades; a theory-led selection of variables, as demonstrated with the DAG in Fig. 1; the use of three different birth outcomes; and disaggregation by both socioeconomic deprivation (particularly pertinent to the hypothesis being explored) and sex of the baby (which has been shown to be important in some analyses of maternal stress and low birthweight [10]).

### Relevance to other studies

There is ample evidence of the impact of different types of maternal stress on adverse birth outcomes. It has been shown for many different types of stressful ‘life events’ including individual factors such as financial problems (discussed further below), stressful employment, the loss of loved ones, separation/divorce, family issues [8], as well as societal stressors such as terrorist attacks (e.g. the September 11th attack in New York in 2001 [35], and the attack in Paris in November 2015 [36]). The causal pathways have also been discussed in detail and include biological processes that affect the hypothalamic–pituitary–adrenal (HPA) axis, changes to normal placental function, and maternal behaviours such as the use of tobacco and alcohol in response to stress and anxiety [7, 10, 11]. Some studies have suggested that male foetuses are more susceptible to these effects [10], something reflected to a small degree in our results, given both the earlier breakpoints identified in the segmented regression, and the slightly higher odds ratios observed in the logistic modelling analyses. Effects have also been shown to be strongest when in relation to both chronic, long-lasting, causes of stress (rather than shorter, isolated, events), and to multiple, simultaneous, stressors [7] which is again potentially relevant here. The evidence regarding the different types of outcome most affected are mixed and can appear inconsistent: some studies show similar effects (e.g. similar socioeconomic trends and inequalities in all three outcomes analysed here [12]), others have highlighted differences (e.g. showing effects on LBW but not PB) [8, 10].

In terms of studies of more specifically *economic* stressors, there is again a large evidence base including various systematic reviews which consistently demonstrate higher risks of many different adverse birth outcomes among those of lower SEP and/or living in more disadvantaged areas [9, 11], as well as both individual [10] and ecological [37] analyses of the effects of unemployment on risk of/rates of low birthweight and preterm babies. Related to this, a number of authors have highlighted the relationship between periods of economic crisis (including, importantly, in countries which had austerity policies imposed on them) and adverse birth outcomes. For example, in an analysis of over 2 million births in Portugal, Kana *et al*. demonstrated a 20%–25% increase in low birthweight births following the introduction of austerity, with associations between that increase and reductions in GDP, health expenditure and social security all highlighted [16]. Similar increases in low birthweight were observed in Iceland in the period following the 2007/08 financial crash, with the increase higher among non-working mothers [21], while analyses in Spain demonstrated increases in SGA [38] and ‘underweight at birth’ [23] associated with the periods of economic crisis and austerity. The same is true of Greece, with different analyses showing increases in LBW [19, 20, 22] and PB [20], including detailed analyses highlighting greater impacts on poorer females [19]. Finally, Rajmil *et al*. analysed the relationship between austerity and different child health outcomes and determinants across 16 European countries, and showed that countries which experienced the highest levels of austerity suffered worse outcomes including higher child poverty and increased rates of LBW [17]. Some of the same authors also undertook a systematic review of the evidence of the impact of austerity on child health, and showed yet more evidence of increases in both LBW and PB associated with the implementation of austerity policies [18].

An alternative interpretation of increasing rates of PB and LBW is that they may simply reflect medical advances meaning that more babies are now likely to survive preterm and/or with a low birthweight. However, this is not supported by the international evidence: excluding multiple births, there has actually been little change in rates of PB and LBW in many European and other high income countries in recent decades (with many showing decreases) (see online appendix for supporting references). Furthermore, as we show here, in Scotland the increases in rates of PB and LBW post-2010 among those living in more deprived neighbourhoods were preceded by sharp decreases from the early 2000s: this also does not support the argument.

The increase in child poverty levels since the early 2010s shown in Fig. 2 has been brought about by vast cuts to the social security budget by the UK Government. Estimates vary, but range from *annual* reductions of around £26 –£56 billion (the initial target set out in 2010 was for an annual cut of £47 billion to have been imposed by 2021). Changes were made to huge numbers of social security payments (there were ∼150 ‘reforms’ made in the first 5 years alone) affecting the poorest in society, particularly families with children. Even more cuts were made to local government funding, resulting in reductions to a wide range of social services, and the poorest areas of the UK were shown to have been most affected [39]. This is relevant as authors have pointed to austerity-related cuts to services, alongside cuts to income, as key to understanding some of the adverse trends in birth outcomes discussed here [16–20, 22, 23, 38].

## Conclusions

Our analyses add to the growing European evidence base of worsening birth outcomes associated with austerity-related periods of economic adversity. Importantly for UK policymakers, we show this for a UK (Scottish) population. Given that austerity policies seem to be continuing under the new Labour government [26], it is vital that policymakers of all political parties in the UK fully understand the evidence of worsening birth trends, their likely causes, and their future implications for child and adult health.

## Supplementary Material

ckae154_Supplementary_Data

## Data Availability

There are no new data associated with this article. Key points Economic ‘austerity’ policies have been shown to have negative impacts on life expectancy and associated population health measures in the UK and other high-income countries. Analyses in mainland European countries have also associated periods of austerity with increasing rates of adverse birth outcomes. We show that the period of UK austerity policies is associated with increased rates of low birthweight and preterm births, particularly among more deprived populations. These changes occurred alongside increases in rates of child poverty. Current and future UK government policymakers need to understand the evidence of the impact of economic policymaking on population health outcomes, particularly among poorer and more vulnerable populations.
