# Supplementary material for: Is the period of austerity in the UK associated with increased rates of adverse birth outcomes?
Source: Eur J Public Health. 2024 Nov 1;34(6):1043–51. doi: 10.1093/eurpub/ckae154 (PMC11631487; doi:10.1093/eurpub/ckae154)
Supplement: ckae154_Supplementary_Data [file ckae154_supplementary_data.pdf]

Appendix

Box A1. Austerity policies in the UK

UK Government 'austerity' measures were first introduced in 2010 (following the 2008/09 'Great Recession') and remain in place today. These were first implemented by the Conservative-Liberal Democrat Coalition Government, and then maintained and expanded by subsequent Conservative administrations over the following 14 years. The policies have been shown to have had a devastating impact on the health of the poorest and most vulnerable populations across all parts of the UK by means of both the loss of important services (including health, social care and other social services) caused by reductions in local government funding, and by increased poverty levels resulting from large-scale, regressive, changes to the social security system[i-iii]. The austerity policies applied to all of the UK and although there were some small differences in their implementation in the devolved nations (Scotland, Northern Ireland and Wales) in comparison with England, the adverse effects of the policies on poorer populations have been shown to be similar[i].

(It is worth noting that the analyses of the impact of UK austerity policies on health outcomes such as life expectancy and age-specific mortality rates have shown that trends changed within approximately two years of implementation (i.e. around 2012 – although this varied slightly across different sections of society)[i,iv,v, xvii]. This is broadly consistent with the results of the segmented regression analyses shown here in our paper).

The 'small differences' referred to above relates to the fact that the impact of some policies were mitigated to a small degree by the devolved governments including in Scotland: cuts to local government funding were less than in England, one housing benefit change (the so-called 'bedroom tax') was effectively reversed[vi-viii], and a new social security benefit has been introduced for low income families with children[ix]. As Figure A5 (further below in this appendix) shows, child poverty rates in Scotland are now lower than in the UK as a whole. It is possible, therefore, that the changes shown in our paper for LBW and PB for the most socioeconomically deprived populations in Scotland

---

[i] McCartney G., Walsh D., Fenton L., Devine R. Resetting the course for population health: evidence and recommendations to address stalled mortality improvements in Scotland and the rest of the UK. Glasgow: Glasgow Centre for Population Health/University of Glasgow; 2022

[ii] Alexiou A, Fahy K, Mason K. et al. Local government funding and life expectancy in England: a longitudinal ecological study. Lancet Public Health 2021; 6(9): e641-e647.

[iii] Seaman R., Walsh D., Beatty C., McCartney G., Dundas R. Social security cuts and life expectancy: a longitudinal analysis of local authorities in England, Scotland, and Wales. Journal of Epidemiology & Community Health 2023; Epub ahead of print: 7 November 2023; doi:10.1136/jech-2023-220328.

[iv] Walsh D., Dundas R., McCartney G., Gibson M., Seaman R. Bearing the burden of austerity: how do changing mortality rates in the UK compare between men and women? Journal of Epidemiology & Community Health 2022; 76: 1027-1033

[v] Fenton L., Minton J., Ramsay J. et al. Recent adverse mortality trends in Scotland: comparison with other high-income countries. BMJ Open 2019; 9: e029936

[vi] Scottish Government. Discretionary Housing Payments to mitigate the Bedroom tax in Scotland. FOI release. Available from: https://www.gov.scot/publications/foi-202000014853

[vii] Welsh Government. Mitigating the impact of the UK Government's welfare reforms. Cardiff: Welsh Government; 2015. Available from: https://www.gov.wales/sites/default/files/publications/2019-05/mitigating-the-impact-of-the-uk-governments-welfare-reforms.pdf

[viii] Northern Ireland Housing Executive (NIHE). Social Sector Size Criteria-Bedroom Tax. Available from: https://www.nihe.gov.uk/housing-help/housing-benefit/social-sector-size-criteria-(bedroom-tax)

[ix] Scottish Government: Scottish Child Payment. See: https://www.gov.scot/policies/social-security/scottish-child-payment/ (Accessed September 2024)

Box A1 – continued

have been even worse in England. However, further research would obviously be required to ascertain whether or not this is the case.

Note that a potential limitation of our paper is that in focussing the analyses on the time periods pre- and post-implementation of austerity policies, we do not distinguish between any potential effects of the 2008/09 recession (which preceded the austerity policies first introduced in 2010) and those of the austerity policies themselves. That said, however, and as Figures 2 (in the main paper) and A5 (further below in this appendix) show, poverty rates increased year on year from 2010 onwards, and a range of analyses have shown clear associations between various austerity policies introduced in that period and multiple facets of poverty (e.g. foodbank use, broader 'food insecurity', homelessness, and more[x-xvi]). Furthermore, other analyses have shown clear negative impacts of austerity policies on health outcomes while adjusting for the effects of economic downturns[xvii].

[x] Loopstra R., Reeves A., Taylor-Robinson D. et al. Austerity, sanctions, and the rise of food banks in the UK BMJ 2015; 350: h1775

[xi] Prayogo E, Chater A, Chapman S, Barker M, Rahmawati N, Waterfall T, Grimble G. Who uses foodbanks and why? Exploring the impact of financial strain and adverse life events on food insecurity. J Public Health 2018; 40(4): 676-683

[xii] Garratt E. Please sir, I want some more: an exploration of repeat foodbank use. BMC Public Health. 2017 Nov 21;17(1):828

[xiii] Sosenko, F., Bramley, G. & Bhattacharjee, A. Understanding the post-2010 increase in food bank use in England: new quasi-experimental analysis of the role of welfare policy. BMC Public Health 2022; 22: 1363

[xiv] Jenkins RH, Aliabadi S, Vamos EP, Taylor-Robinson D, Wickham S, Millett C, Laverty AA. The relationship between austerity and food insecurity in the UK: A systematic review. EClinicalMedicine 2021; 33: 100781

[xv] Loopstra R., Reeves A., Barr B., Taylor-Robinson D., McKee M., Stuckler D. The impact of economic downturns and budget cuts on homelessness claim rates across 323 local authorities in England, 2004–12. Journal of Public Health 2016; 38 (3): 417–425

[xvi] Fetzer T., Sen S., Souza P. C.L. Housing insecurity and homelessness: evidence from the UK. Journal of the European Economic Association 2023; 21 (2): 526-559

[xvii] McCartney G, McMaster R, Popham F, Dundas R, Walsh D. Is austerity a cause of slower improvements in mortality in high-income countries? A panel analysis. Social Science & Medicine 2022; 313: 115397

Box A2. Descriptions of outcome variables

- Low birthweight (LBW) babies are defined as the percentage of all live births that weigh <2,500g.
- Premature births (PB) are defined as the percentage of all live births born before 37 weeks' gestation;
- Small-for-gestational-age (SGA) babies are defined here as the percentage of all live births with a birthweight <3rd percentile of the sex and gestation specific distribution of the 1990 UK population; this is taken from the UK -World Health Organisation (WHO) growth charts – the 3rd percentile cut-off has been used previously as a proxy for foetal growth restriction[xviii].

LBW babies are defined as such *regardless of gestational age*. A large proportion, but not all, will be LBW as a result of being preterm and not having enough time to grow; thus, there is a clear overlap between these two categories of birth outcome.

SGA babies are those who are smaller than would be expected given the amount of time they have had to grow. For some, this may be the result of constitutional factors such as maternal height and weight; however, in many cases it may be due to restricted growth as a result of factors such as poor placental function (hence our focus on that group in our cut-off).

Babies born below the 3rd centile for weight will also be LBW if they are born below around 38 to 39 weeks gestation (depending on sex. Above this gestation they may be SGA but not LBW. Trends in SGA and LBW may differ if rates of LBW are largely driven by changes in PB - i.e. caused by babies having less time to grow, rather than being of lower weight than their gestational age would predict.

[xviii] Jardine J., Walker K., Gurol-Urganci I., Webster K. et al. Adverse pregnancy outcomes attributable to socioeconomic and ethnic inequalities in England: a national cohort study. Lancet 2021; 398(10314): 1905-1912

Figure A1. Directed acyclic graph (DAG) illustrating the causal pathways between exposure (austerity) and outcome (premature birth (PB), low birthweight (LBW), small for gestational age (SGA) birth).

Note that this is a deliberately simplified DAG, developed initially for SGA (as a proxy for foetal growth restriction), and with the assumption that the causal pathways between austerity and preterm birth (PB) and low birthweight (LBW) are similar.

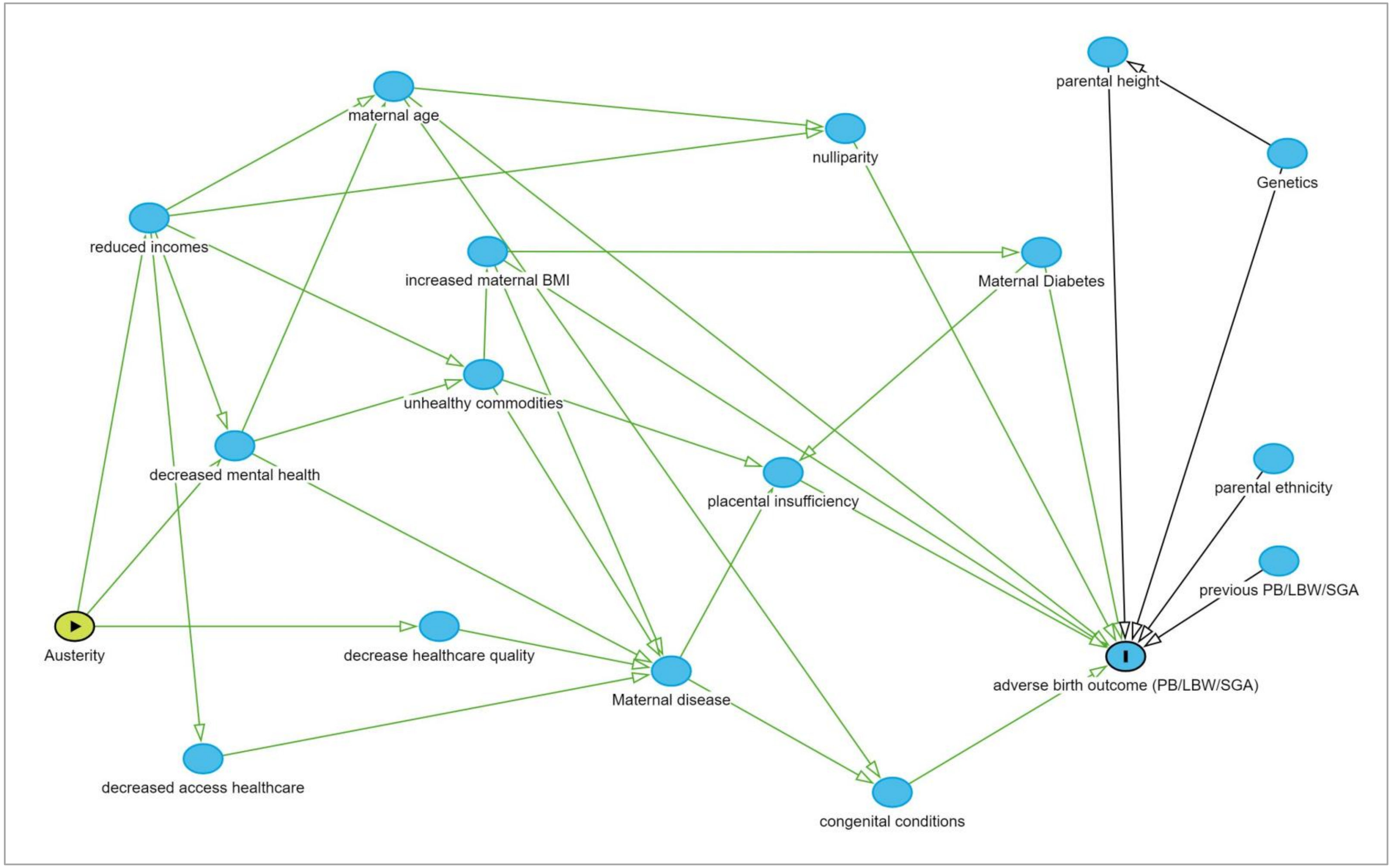

Figure A2. Trends in (a) premature births, (b) low birthweight babies, (c) small for gestational age babies by deprivation quintile, Scotland 1981/83 – 2017/19

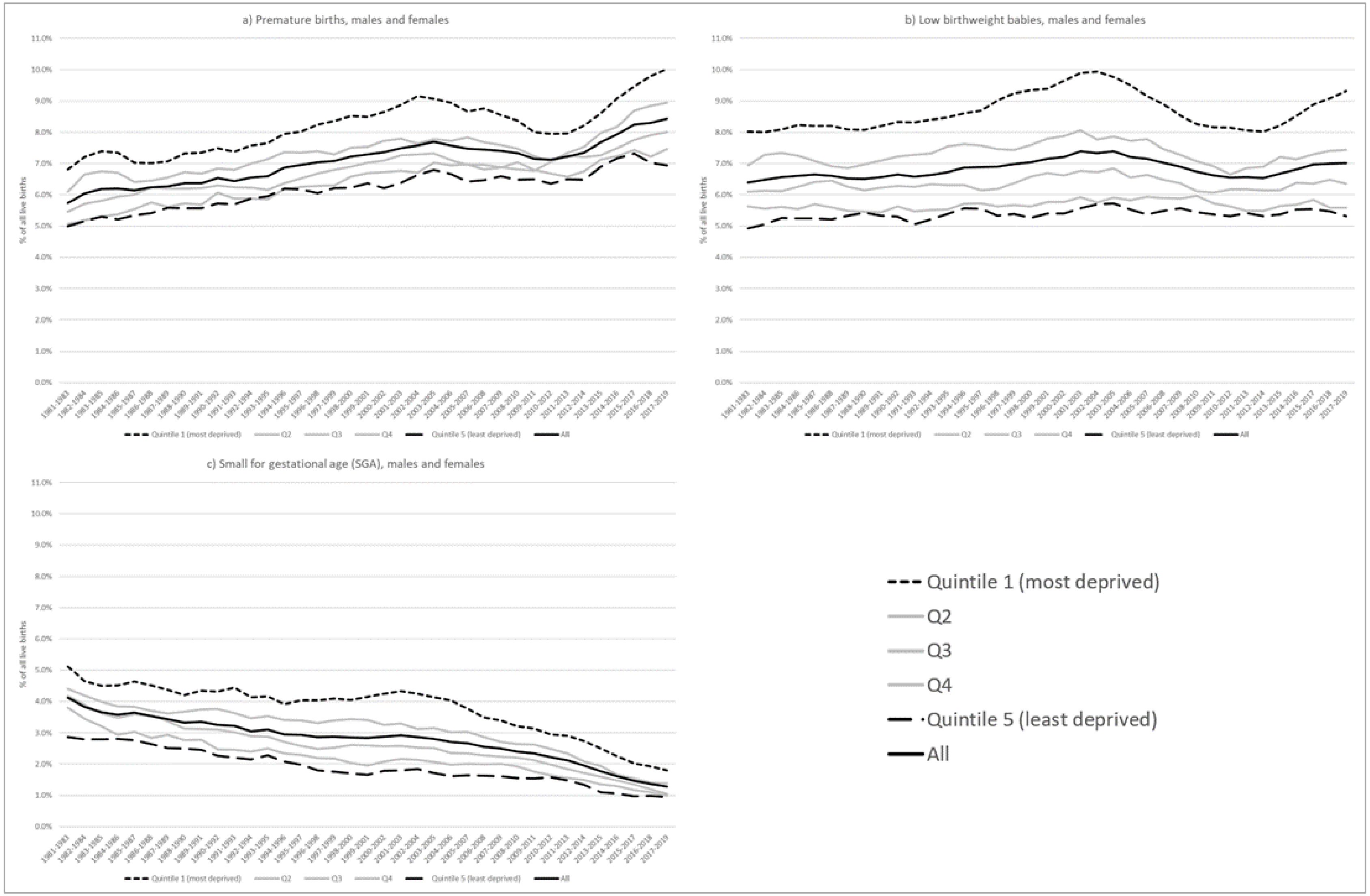

Figure A3. Trends in (a) premature births, (b) low birthweight babies, (c) small for gestational age babies, Scotland and its most and least deprived quintiles, 1981/83 – 2017/19 – MALES

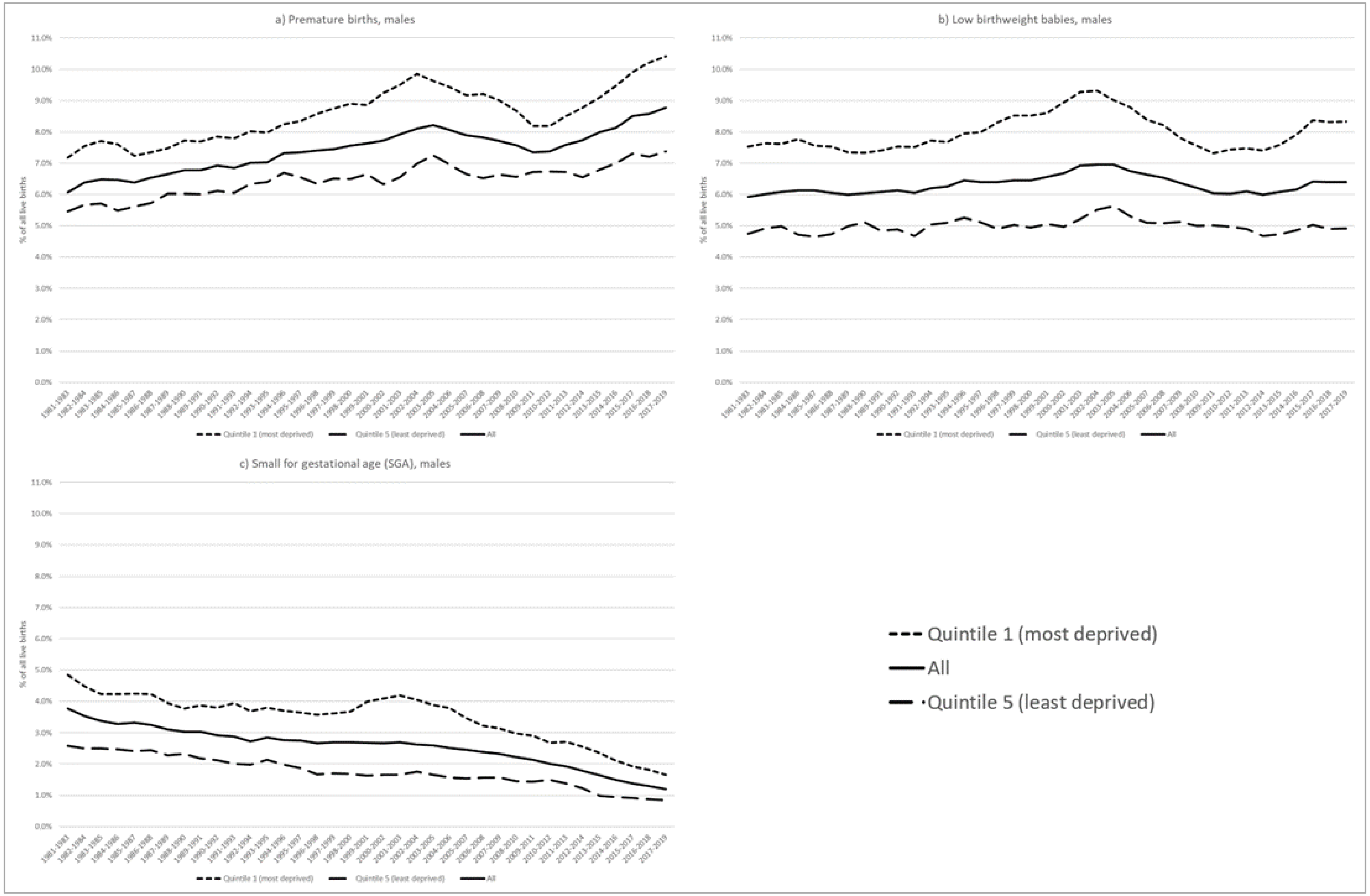

Figure A4. Trends in (a) premature births, (b) low birthweight babies, (c) small for gestational age babies, Scotland and its most and least deprived quintiles, 1981/83 – 2017/19 – FEMALES

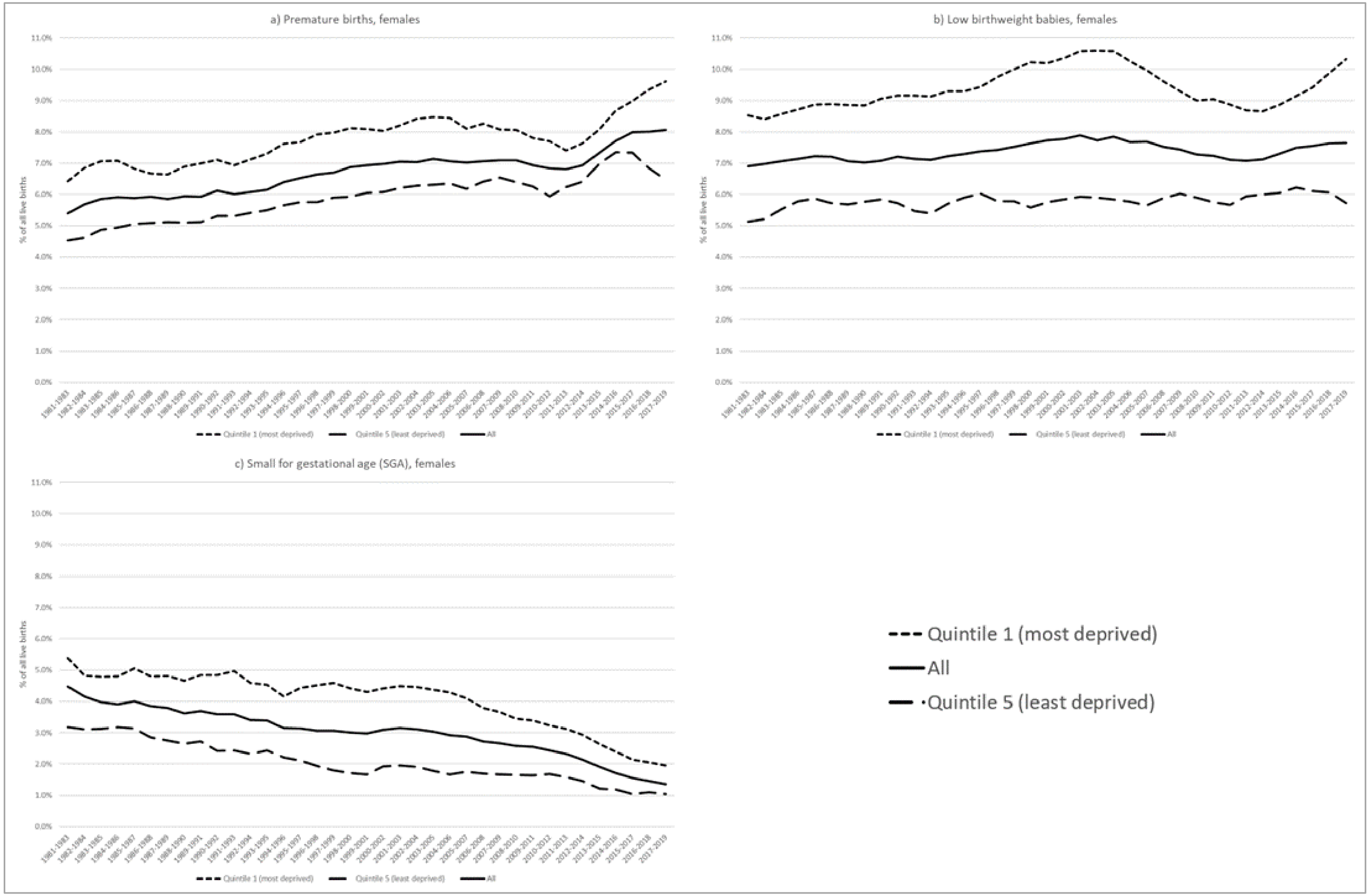

Figure A5. Child poverty rates, UK and Scotland, Scotland 1981-2019

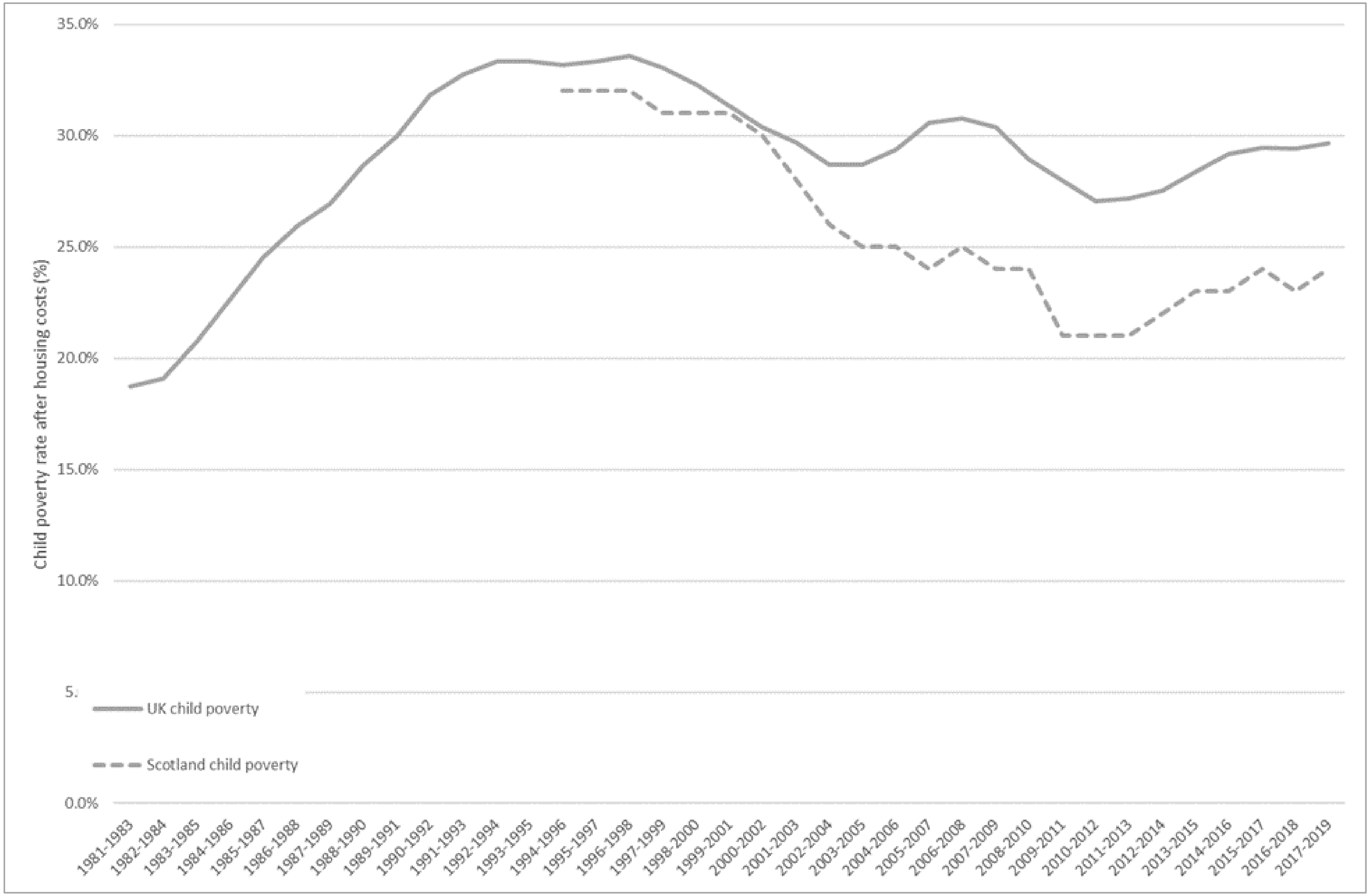

Figure A6. Trends in premature births, Scotland and its most and least deprived quintiles, 1981/83 – 2017/19, shown as percentages of (a) all live births (b) all live singleton births and (c) all live singleton births excluding planned Caesarean sections.

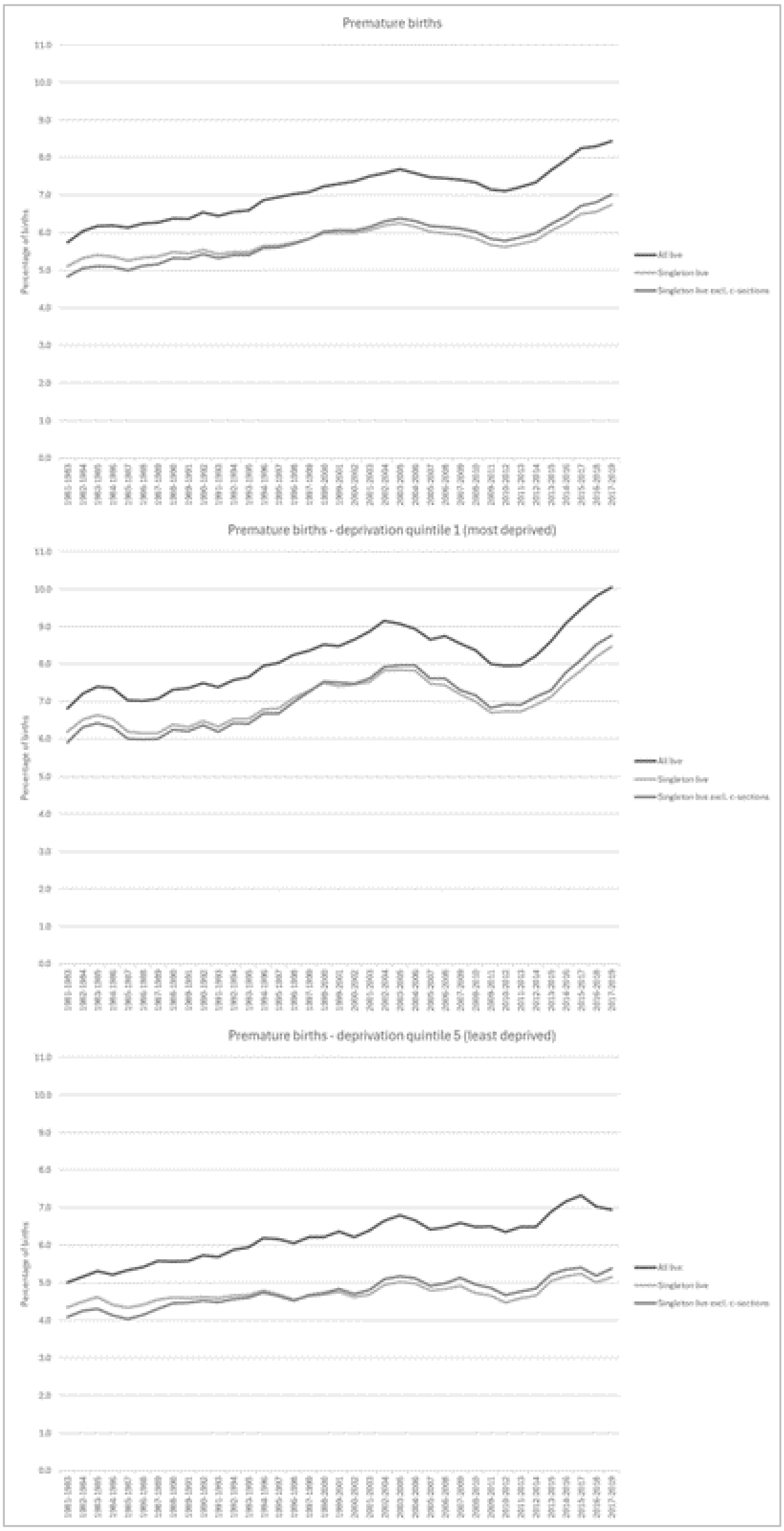

Figure A7. Trends in low birthweight births, Scotland and its most and least deprived quintiles, 1981/83 – 2017/19, shown as percentages of (a) all live births (b) all live singleton births and (c) all live singleton births excluding planned Caesarean sections.

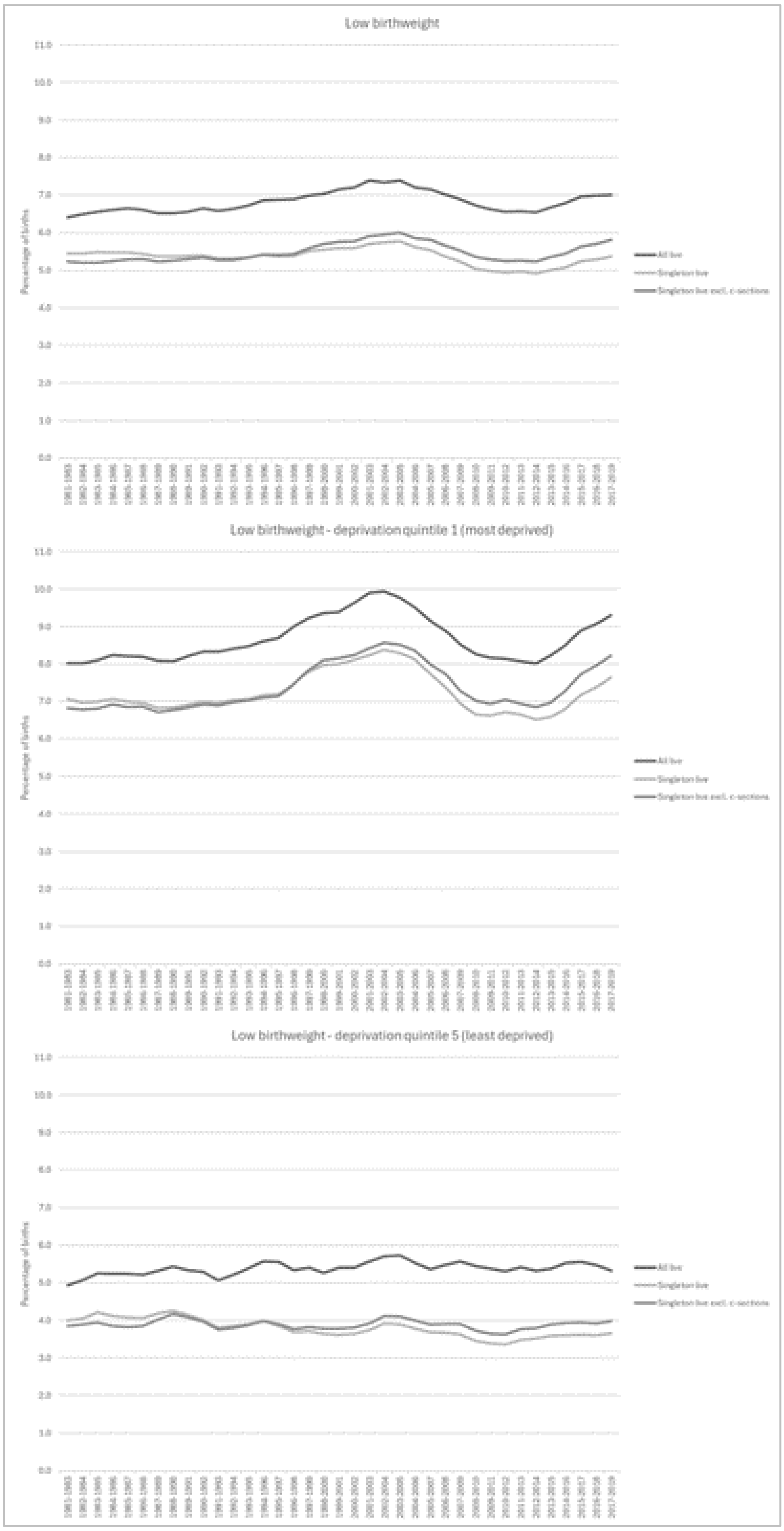

Figure A8. Segmented Regression (with multiple breakpoints) plots: three main outcomes.

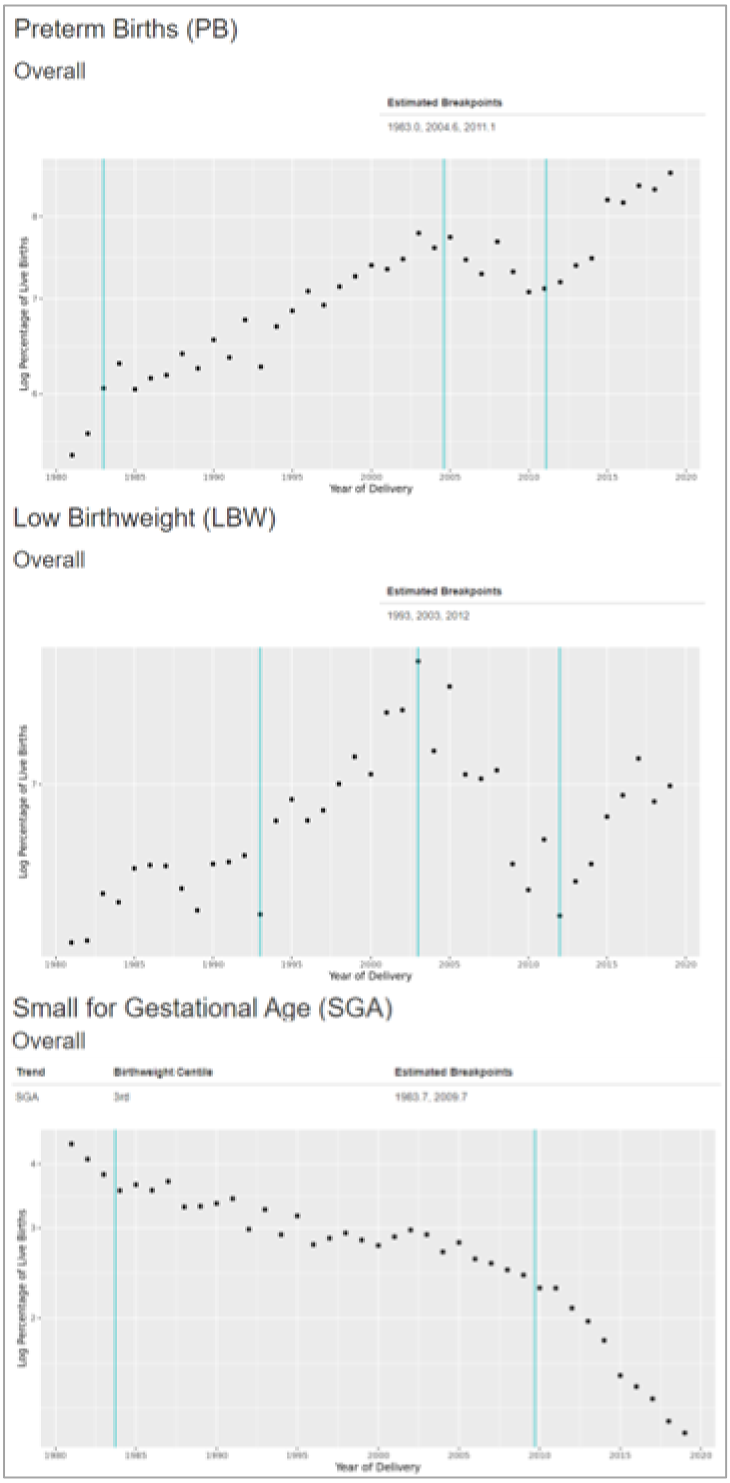

Figure A9. Segmented Regression (with multiple breakpoints) plots: premature birth stratified by deprivation quintile and sex.

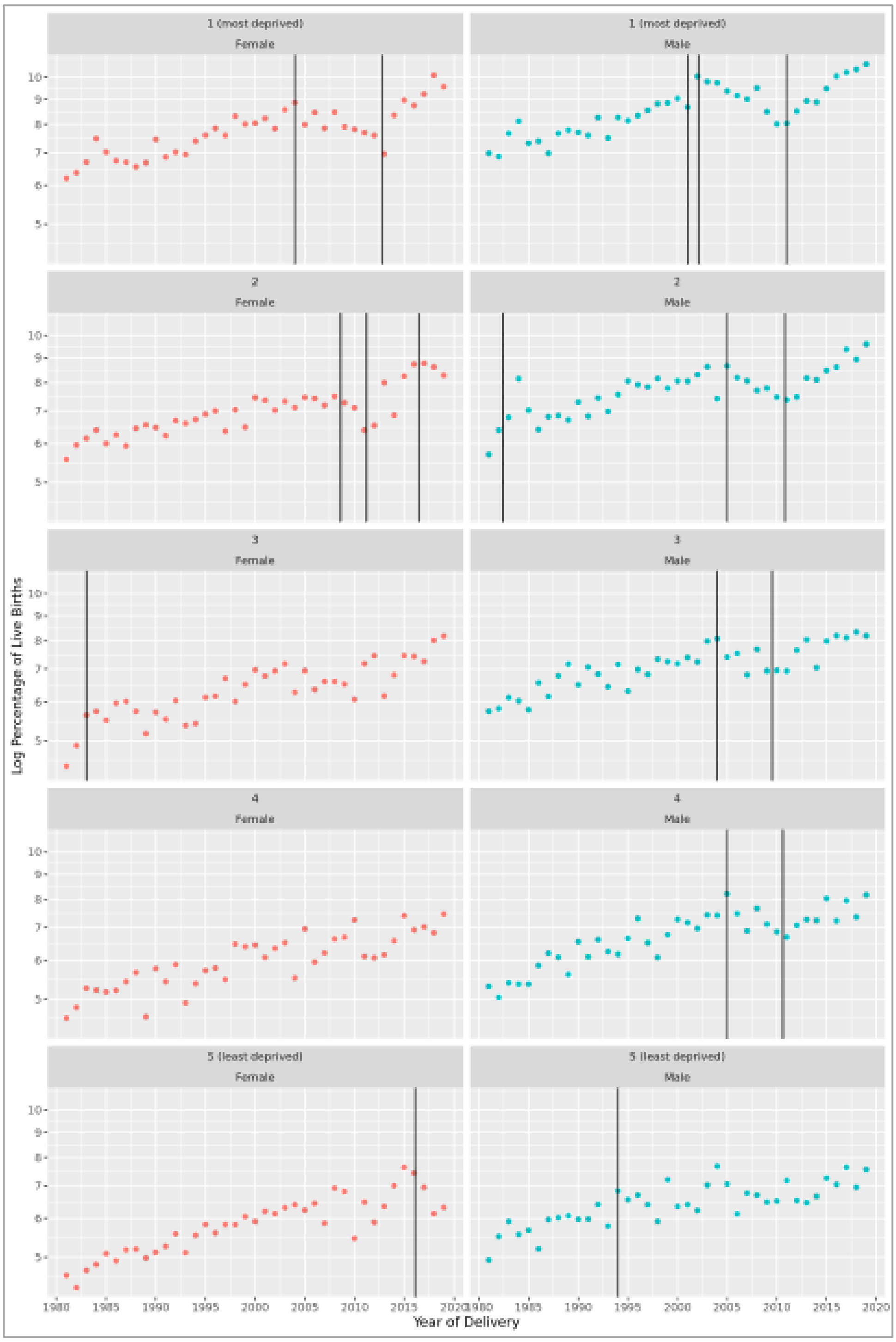

Figure A10. Segmented Regression (with multiple breakpoints) plots: low birthweight stratified by deprivation quintile and sex.

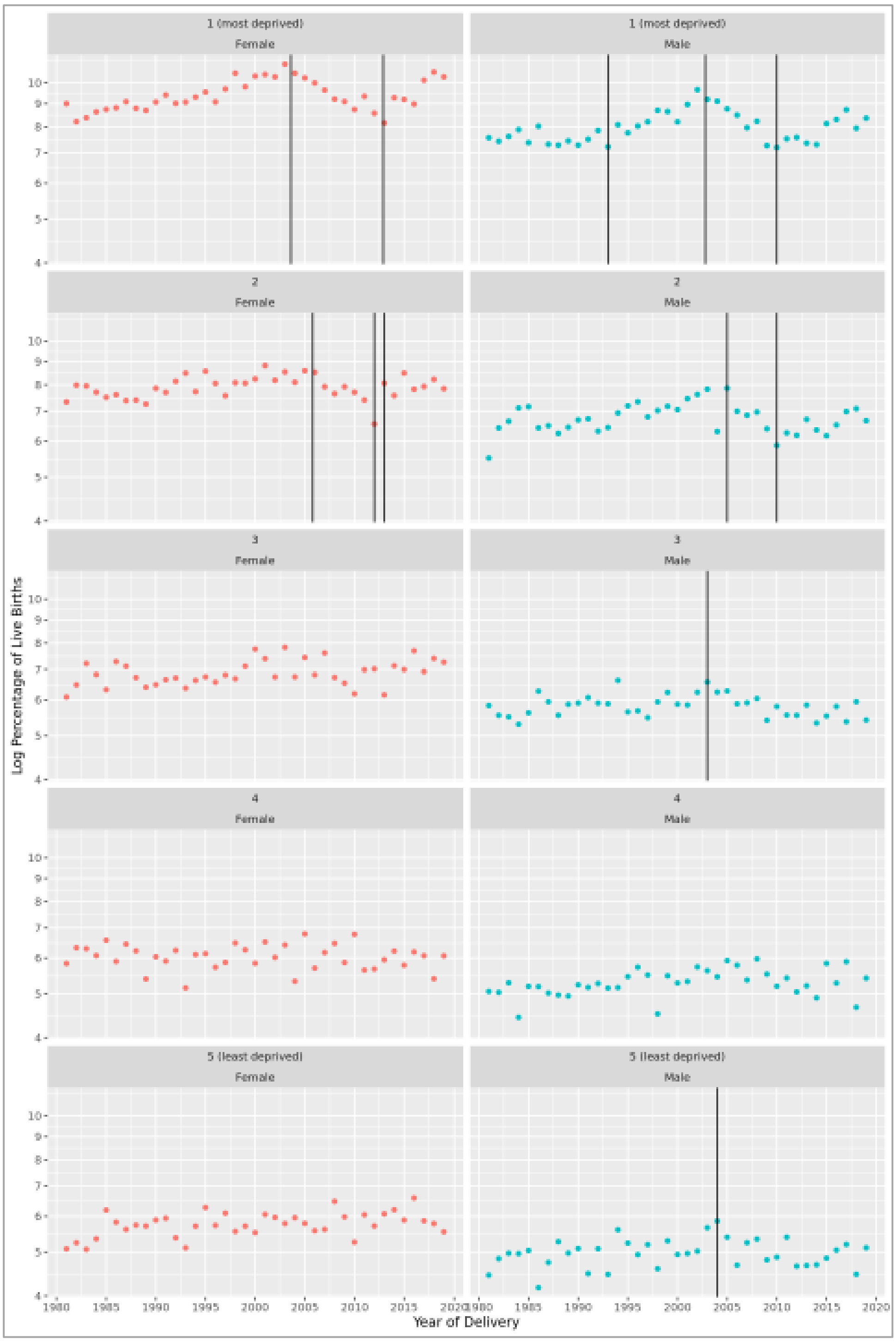

Figure A11. Segmented Regression (with multiple breakpoints) plots: small for gestational age (SGA) stratified by deprivation quintile and sex.

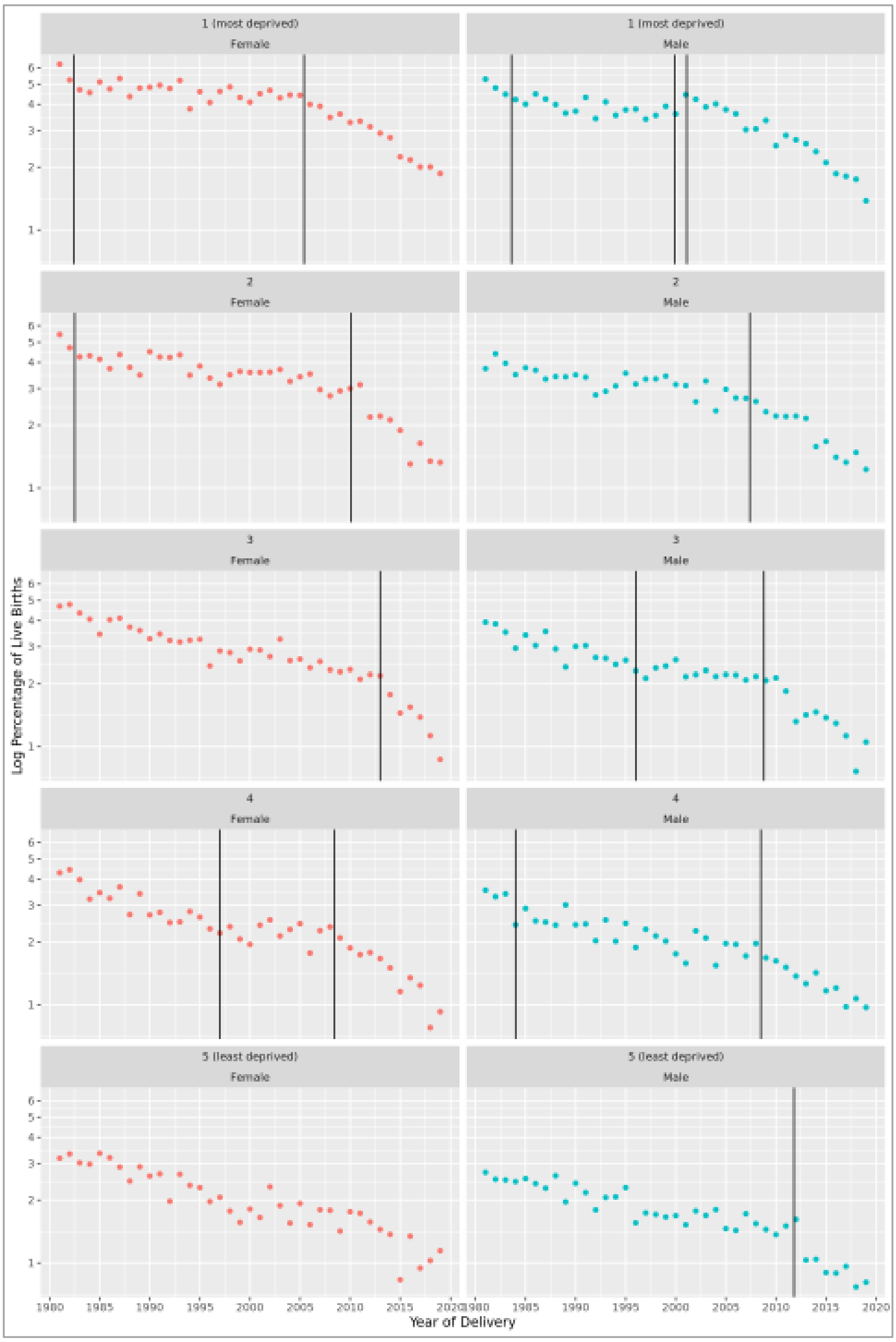

Box A1. Further details of segmented regression modelling.

Let $y_i = 0{,}1$ denote the outcome that baby i is born with the outcome of interest (ie prematurely, of low birthweight, or less than 3rd centile), such that $y_i \sim Bin(1,\ p_i), i = 1, \ldots, N$.

The probability that baby i is born under with the outcome is $p_i = invlogit(eta_i)$ where;

$$eta_i = \beta_0 + year_i * \beta_1 + I_1(year_i) * \delta_1 + \ldots + I_k(year_i) * \delta_k + \epsilon_i$$

- k is the number of breakpoints
- β_0 is the intercept
- β_1 is the slope estimate before the breakpoint
- $year_i$ is the year that baby i was delivered
- $I_k(year_i) = \begin{cases} 0 \ if \ year_i \ < \tau_k \\ year_i - \tau_k \ if \ year_i >= \tau_k \end{cases}$
- τ_k is the location of the estimated breakpoint
- δ_k is the slope estimate after the breakpoint
- ϵ_i is the error term

Box A2. Further details of logistic regression modelling.

Let $y_i = 0{,}1$ denote the outcome that baby i is born with the outcome of interest, such that $y_i \sim Bin(1,\ p_i), i = 1, \ldots, N$.

The probability that baby i is born with the outcome is $p_i = invlogit(eta_i)$ where;

$$eta_i = \beta_0 + year_i * \beta_1 + I(year_i) * \delta + height_i * \beta_2 + \epsilon_i$$

- β_0 is the intercept
- β_1 is the slope estimate before austerity
- $year_i$ is the year that baby i was delivered
- $I(year_i) = \begin{cases} 0\ if\ year_i\ < 2010 \\ year_i - 2010\ if\ year_i >= 2010 \end{cases}$
- δ is the change in slope after austerity
- β_2 is the coefficent of height
- $height_i$ is the height of baby i's mother
- ϵ_i is the error term

Additional references for article

As the journal has a maximum number of 40 references, some had to be excluded from the main manuscript, but are included here.

Introduction:

- evidence of changes to population health in the UK since the early 2010s and the causal role of UK Government austerity policies: [1-17]
- The three birth outcomes (PB, LBW and SGA) are associated with poorer outcomes later in life: [18-20]

Methods

- Public Health Scotland's Maternity Inpatient and Day Case Scottish Morbidity Record dataset (SMR02): [21]
- World Health Organisation (WHO) UK growth charts: [22]
- "ljr" package of R: [23]

Discussion

- Trends in PB and LBW in European and other high income countries in recent decades[24-26]
- Scale of reductions to social security budget:[27,28].
- 150 reforms to social security payments and eligibility: [29]
- Impact of reforms greatest on families and children: [30,31]
- Poorest areas of the UK were shown to have been most affected: [31,32]

These references:

[1] McCartney G., Walsh D., Fenton L., Devine R. Resetting the course for population health: evidence and recommendations to address stalled mortality improvements in Scotland and the rest of the UK. Glasgow: Glasgow Centre for Population Health/University of Glasgow; 2022

[2] Walsh D., McCartney G., Minton J., Parkinson J., Shipton D., Whyte B. Changing mortality trends in countries and cities of the UK: a population-based trend analysis. BMJ Open 2020; 10: e038135

[3] Currie J, Boyce T, Evans L, Luker M et al. Life expectancy inequalities in Wales before COVID-19: an exploration of current contributions by age and cause of death and changes between 2002 and 2018. Public Health. 2021 Mar 15;193:48-56.

[4] Rashid T, Bennett JE, Paciorek CJ, et al. Life expectancy and risk of death in 6791 communities in England from 2002 to 2019: high-resolution spatiotemporal analysis of civil registration data. Lancet Public Health 2021; 6(11): e805-e816.

[5] Walsh D., Wyper G., McCartney G. Trends in healthy life expectancy in the age of austerity. Journal of Epidemiology & Community Health 2022; 76: 743-745

[6] Goldblatt P. Health Inequalities, Lives Cut Short. London: UCL Institute of Health Equity; 2024. Available from: https://www.instituteofhealthequity.org/resources-reports/health-inequalities-lives-cut-short (Accessed February 2024)

[7] Zhang A., Gagne T., Walsh D., Ciancio A., Proto E., McCartney G. Trends in psychological distress in Great Britain, 1991-2019: evidence from three representative surveys. Forthcoming.

[8] Walsh D., McCartney G. Changing mortality rates in Scotland and the UK: an updated summary. Glasgow: Glasgow Centre for Population Health; 2023

[9] United Nations (UN) Human Rights Council. Visit to the United Kingdom of Great Britain and Northern Ireland: Report of the Special Rapporteur on extreme poverty and human rights. New York: UN; 2019

[10] Stuckler D., Reeves A., Loopstra R., Karanikolos M., McKee M. Austerity and health: the impact in the UK and Europe. Eur J Public Health. 2017; 27(suppl_4):18–21.

[11] Taylor-Robinson D, Whitehead M, Barr B. Great leap backwards. BMJ 2014; 349: g7350

[12] Alexiou A, Fahy K, Mason K. et al. Local government funding and life expectancy in England: a longitudinal ecological study. Lancet Public Health 2021; 6(9): e641-e647.

[13] Richardson E, Fenton L, Parkinson J, Pulford A, Taulbut M, McCartney G, Robinson M. The effect of income-based policies on mortality inequalities in Scotland: a modelling study. Lancet Public Health 2020: e150-e156..

[14] Martin S., Longo F., Lomas J., Claxton K. Causal impact of social care, public health and healthcare expenditure on mortality in England: cross-sectional evidence for 2013/2014. BMJ Open 2021; 11(10): e046417

[15] Seaman R., Walsh D., Beatty C., McCartney G., Dundas R. Social security cuts and life expectancy: a longitudinal analysis of local authorities in England, Scotland, and Wales. Journal of Epidemiology & Community Health 2023; Epub ahead of print: 7 November 2023; doi:10.1136/jech-2023-220328.

[16] McCartney G, McMaster R, Popham F, Dundas R, Walsh D. Is austerity a cause of slower improvements in mortality in high-income countries? A panel analysis. Social Science & Medicine 2022; 313: 115397

[17] Broadbent P., Walsh D., Katikireddi S.V., Gallagher C., Dundas R., McCartney G. Is austerity responsible for the stalled mortality trends across many high income countries? A systematic review. International Journal of Social Determinants of Health and Health Services 2024; doi:10.1177/27551938241255041

[18] de Mendonça ELSS, de Lima Macêna M, Bueno NB, de Oliveira ACM, Mello CS. Premature birth, low birth weight, small for gestational age and chronic non-communicable diseases in adult life: A systematic review with meta-analysis. Early Hum Dev. 2020; 149: 105154.

[19] De Mola C., De França G., De Avila Quevedo L., Horta, B. Low birth weight, preterm birth and small for gestational age association with adult depression: Systematic review and meta-analysis. British Journal of Psychiatry 2014; 205(5): 340-347

[20] Black S.E., Devereux P.J, Salvanes K.G., From the Cradle to the Labor Market? The Effect of Birth Weight on Adult Outcomes. The Quarterly Journal of Economics 2007; 122 (1): 409–439

[21] Public Health Scotland. National data sets. Available from: https://publichealthscotland.scot/services/national-data-catalogue/national-datasets/a-to-z-of-datasets/maternity-inpatient-and-day-case-scottish-morbidity-record-smr02 (Accessed February 2024)

[22] Cole T.J., Freeman J.V., Preece M.A. British 1990 growth reference centiles for weight, height, body mass index and head circumference fitted by maximum penalized likelihood. Stat Med. 1998; 17(4): 407-29

[23] Czajkowski M., Gill R., Rempala G. Package 'ljr'. Available from Comprehensive R Archive Network (CRAN) website: https://cran.r-project.org/web/packages/ljr/ljr.pdf (Accessed March 2024).

[24] Zeitlin J., Szamotulska K., Drewniak N. et al. Preterm birth time trends in Europe: a study of 19 countries. BJOG 2013; 120: 1356–1365.

[25] Okwaraji Y.B., Krasevec J., Bradley E. et al. National, regional, and global estimates of low birthweight in 2020, with trends from 2000: a systematic analysis. The Lancet 2024; 403 (10431): 1071-108

[26] Chawanpaiboon S, Vogel JP, Moller A-B, Lumbiganon P, Petzold M, Hogan D, et al. Global, regional, and national estimates of levels of preterm birth in 2014: a systematic review and modelling analysis. The Lancet. Global health. 2019;7(1): e37–e46

[27] De Henau J. Gender impact of social security spending cuts. London: Women's Budget Group, 2017

[28] Scottish Government. Welfare Reform (Further Provision) (Scotland) Act 2012: annual report 2017. Edinburgh: Scottish Government, 2017

[29] Office for Budget Responsibility (OBR). Welfare trends report. London: OBR; 2016.

[30] Portes J., Reed H. The cumulative impact of Tax and welfare reforms. Manchester: Equality and Human Rights Commission (EHRC); 2018.

[31] Beatty C., Fothergill S. The uneven impact of welfare reform: the financial losses to places and people. Sheffield: Sheffield Hallam; 2016.

[32] Gray M., Barford A. The depths of the cuts: the uneven geography of local government austerity. Cambridge Journal of Regions, Economy and Society 2018; 11: 541–63
